# Synergistic effect of PbS nanoparticle deposition on TiO_2_ nanotubes for efficient indoor air remediation

**DOI:** 10.1039/d5ra06532a

**Published:** 2025-10-20

**Authors:** Mabrouk Abidi, Safa Jemai, Achraf Amir Assadi, Anouar Hajjaji, Mounir Gaidi, Amine Aymen Assadi, Nabisab Mujawar Mubarak, My Ali El Khakani

**Affiliations:** a Laboratoire de Photovoltaïque, Centre de Recherches et des Technologies de Energie Technopôle de Borj-Cédria, BP 95 Hammam-Lif 2050 Tunis Tunisia; b Center for Research on Microelectronics and Nanotechnology CRMN Sousse Techno Park, Sahloul, BP 334 4054 Sousse Tunisia; c Center of Advanced Research Materials, Research Institute of Sciences and Engineering, University of Sharjah Sharjah P.O. Box 27272 United Arab Emirates; d Deparatment of Chemical Engineering, College of Engineering, Imam Mohammad Ibn Saud Islamic University (IMSIU) 11432 Riyadh Saudi Arabia Aaassadi@imamu.edu.sa; e Institut National de La Recherche Scientifique (INRS), Centre Énergie Matériaux et Télécommunications (INRS-EMT) 1650 Boulevard Lionel Boulet Varennes QC J3X 1P7 Canada

## Abstract

This study investigated the photocatalytic performance of PbS-NPs/NTs-TiO_2_ photocatalysts for the degradation of Butane-2, 3-Dione (BUT). The impact of decorating dioxide nanotubes (TiO_2_-NTs) with lead sulfide nanoparticles (PbS-NPs) on the degradation of volatile organic compounds (VOCs) was the focus of the investigation. The titanium dioxide nanotubes (TiO_2_-NTs) were synthesized by electrochemical anodization of a titanium-based substrate. At the same time, lead sulfide nanoparticles (PbS-NPs) were deposited using the pulsed laser deposition (PLD) technique. The mean diameter of PbS-NPs increased from approximately 10 nm to 20 nm as we modified the laser ablation pulse number (*N*_LP_) from 500 to 10 000. The cubic crystalline phase of PbS nanoparticles was revealed by X-ray diffraction analysis. An increase in the number of laser ablation pulses led to PbS-NPs aggregation, as indicated by observations in scanning electron microscopy (SEM). The PbS surface exhibited a compact and uniform morphology with strong adherence to the substrate, as demonstrated by Atomic Force Microscopy (AFM) analysis. The roughness (*R*_ms_) transitioned from 52 nm for pure TiO_2_-NTs to 111 nm for *N*_LP_ = 5000, decreasing to 22 nm for *N*_LP_ = 10 000. Photoluminescence (PL) spectra showed that lower PL intensity was exhibited by PbS-NPs/TiO_2_-NTAs compared to pure NTs, with the lowest PL intensity observed for *N*_LP_ = 5000. Absorbance spectra of TiO_2_-NTAs adhered to quartz substrates revealed a calculated band gap of 3.1 eV for TiO_2_. The nanocomposite demonstrated high photocatalytic performance in BUT removal when the PbS nanoparticles were deposited at an optimal laser pulse count of *N*_LP_ = 5000. Results indicated that superior BUT adsorption and degradation capabilities were possessed by the PbS-NPs-modified TiO_2_-NTs when compared to pure TiO_2_-NTs. Notably, the highest photocatalytic activity with an efficiency exceeding 75% was exhibited by PbS-NPs/TiO_2_-NTs-5000.

## Abstract 2

This study investigated the photocatalytic performance of PbS-NPs/NTs-TiO_2_ photocatalysts for the degradation of Butane-2, 3-Dione (BUT).

## Graphical abstract

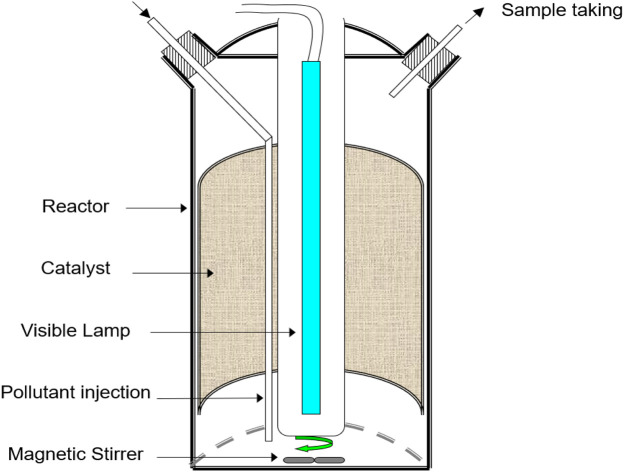

## 1 Introduction

Nanotechnological advancements over the past two decades have enabled precise control of materials at the nanoscale. This achievement has facilitated miniaturization and induced changes in material properties, resulting in novel physicochemical characteristics that differ from those at the macroscopic scale. This breakthrough has opened various applications across various technological domains.

Numerous studies have been dedicated to exploring TiO_2_ tubular nanostructures due to their unique surface properties,^1^ exceptional biocompatibility, and uniformity. The fabrication of nanostructured titania can be achieved through a variety of methods, including metalorganic chemical vapor deposition,^2^ sol–gel synthesis,^3^ hydrothermal techniques,^4^ and the anodization of titanium.^5–11^ Among these methods, our primary focus revolves around the anodization of titanium foil, a process that enables the creation of self-organized TiO_2_ nanotube arrays. This approach precisely controls geometric parameters such as length, diameter, and wall thickness by selecting appropriate anodization parameters. The ability to fine-tune the dimensions of these nanotube arrays enhances their versatility for applications in diverse fields, including dye-sensitized solar cells,^12^ biosensors,^13,14^ and highly efficient photocatalytic systems.^15–17^

One significant challenge associated with using TiO_2_ in various applications is its optical absorption range and the rapid recombination of photogenerated electron–hole pairs. TiO_2_, as a photocatalyst, is characterized by a wide band gap (3.2 eV) semiconductor that predominantly absorbs light in the ultraviolet range, constituting only 4% of the solar spectrum.^18^ This inherent limitation substantially hinders these photocatalysts' efficiency in directly harnessing solar energy. Consequently, it is critical to devise effective strategies to enhance charge separation efficiency and the photoactivity of titania nanotubes. Such strategies include doping with metals (*e.g.*, Ag,^19–22^ Cu, Au), decorating with metallic nanoparticles or semiconductors (*e.g.*, PbS,^23,24^ Cu_2_O^25,26^), or surface decoration with nanostructured noble metals (*e.g.*, Pt,^27^ Pd,^28^ Ru) to serve as catalysts.

Eliminating volatile organic compounds (VOCs) represents a fundamental application of TiO_2_-NTs. A wide range of VOCs, encompassing ketones, aldehydes, aromatics, and mercaptans, are routinely released into the atmosphere through industrial and agricultural processes, animal husbandry, and fertilizer applications,^29,30^ constituting a substantial source of harmful airborne pollutants. These hazardous emissions severely threaten human health and the environment,^29–31^ underscoring the necessity for mitigating their release. To reduce this environmental problem, researchers have used a wide range of treatment methods, including adsorption,^32^ thermal and catalytic incineration,^33^ and bio-filtration.^34^ As a result, substantial research efforts have been dedicated to developing innovative and cost-effective technologies for efficiently treating VOCs.^30^

In this context, the present study differs significantly from our previous work, which primarily focused on liquid-phase dye degradation,^23^ by specifically addressing the VOCs remediation in t gas-phase. This transition is crucial because mass transfer mechanisms in the gas phase (diffusion in air) differ fundamentally from those in the liquid phase. In addition, Langmuir–Hinshelwood kinetics and competitive adsorption effects with atmospheric components (O_2_, H_2_O) are specific to the gaseous environment. Our research focuses on evaluating the photocatalytic performance of PbS-NPs/TiO_2_-NTs nanocomposites for the adsorption and degradation of VOCs, using butane-2,3-dione as a representative model for cheese odors. It should be noted that, to our knowledge, previous studies have not explored the catalytic activity of PbS-NPs/TiO_2_-NTs prepared by pulsed laser deposition (PLD) for the detection and removal of VOCs from indoor air. The 75% degradation efficiency achieved for butane-2,3-dione at concentrations that conform to indoor air quality standards demonstrates significant practical applicability for real-world air purification and ventilation systems, representing a substantial advance over our previous work and addressing a distinct and urgent environmental challenge.

## 2 Materials and methods

We prepared the samples using 99.99% pure titanium (Ti) plates with dimensions of 2 × 1.5 cm^2^ and a thickness of approximately 0.5 mm. To ensure consistency, all Ti plates possessed identical characteristics. Initially, the samples were polished using abrasive papers of varying grain sizes ranging from 320 to 2000. To activate the surface and prevent adhesion issues, we sequentially rinsed the samples with acetone, ethanol, and bi-distilled water for 10 minutes each in an ultrasonic bath. This process eliminated impurities and foreign substances introduced during the polishing phase. After treatment, we allowed the samples to air-dry for a specific duration. We conducted electrochemical experiments at room temperature within the laboratory setting. The anodic oxidation of the samples was performed under the same experimental conditions in an electrolyte cell containing 100 mL of ethylene glycol, 2% water (H_2_O), and 1% ammonium fluoride (NH_4_F). The anodization process lasted 120 minutes at a fixed voltage of 60 V and a temperature of approximately 25 °C. To achieve the anatase crystalline form of TiO_2_ nanotubes, crucial for photocatalytic solid activity, we calcined the obtained samples hour at 400 °C.

For the deposition of PbS thin films, a KrF excimer laser (wavelength = 248 nm) operating at a repetition frequency of 20 Hz was directed at a 45° angle onto the rotating PbS target (99.995% purity). A laser power of 200 mJ was employed to guarantee uniform film thickness. The deposition of PbS thin films was carried out on two types of substrates, specifically conventionally cleaned Si and quartz. The nanotubes substrates were positioned on a substrate holder parallel to the target, maintaining a perpendicular distance of 8 cm. The depositions occurred at room temperature under a helium gas environment at a pressure of approximately 400 mTorr. Before deposition, the PbS target was systematically cleaned *via* laser ablation for 10 minutes, with the substrate shielded from the ablation plume by a shutter. The nominal thickness of the PbS film varied between 15 nm and 300 nm by adjusting the number of laser ablation pulses from 500 to 10 000. The determination of the nominal PbS film thickness (*t*) was based on the multiplication of the number of laser pulses by the average deposition rate (nanometer/pulse), as determined from the deposition of a sufficiently thick film (150 nm) whose thickness was directly measured from cross-section scanning electron microscopy observations. The manipulation of the number of laser pulses facilitated the control of the deposition thickness, as outlined in Table 1.

**Table 1:** Thickness variation of the PbS layer with the number of laser pulses

Number of pulses	1000	5000	10 000
Thickness (nm)	30	150	300

The crystallographic structure analysis was conducted using the X-ray diffraction (XRD) technique, employing a Philips X'pert-MPD X-ray diffractometer with Cu Kλ radiation in a grazing incidence configuration. To explore the surface morphology and nanostructure of the treated surfaces; we employed multiple techniques, including atomic force microscopy (AFM), scanning electron microscopy (SEM), and transmission electron microscopy (TEM). These structural characterizations provided insights into nanoparticle distribution, surface roughness, and crystallinity, which are closely related to the optical behavior of the samples.

To determine the band gap energy of TiO_2_ nanotubes, we analyzed the optical transmittance spectra (*T*(*λ*)) measured on films directly deposited onto quartz substrates. Spectrophotometric measurements of *T*(*λ*) and reflectance (*R*(*λ*)) were conducted across a wavelength range of 250 to 1200 nm using a PerkinElmer Lambda 950 spectrophotometer equipped with an integrating sphere. Photoluminescence (PL) properties were assessed using an Ar laser (*λ* = 488 nm) and detected by a cooled GaInAs detector utilizing a standard lock-in technique.

**Fig. 1:**
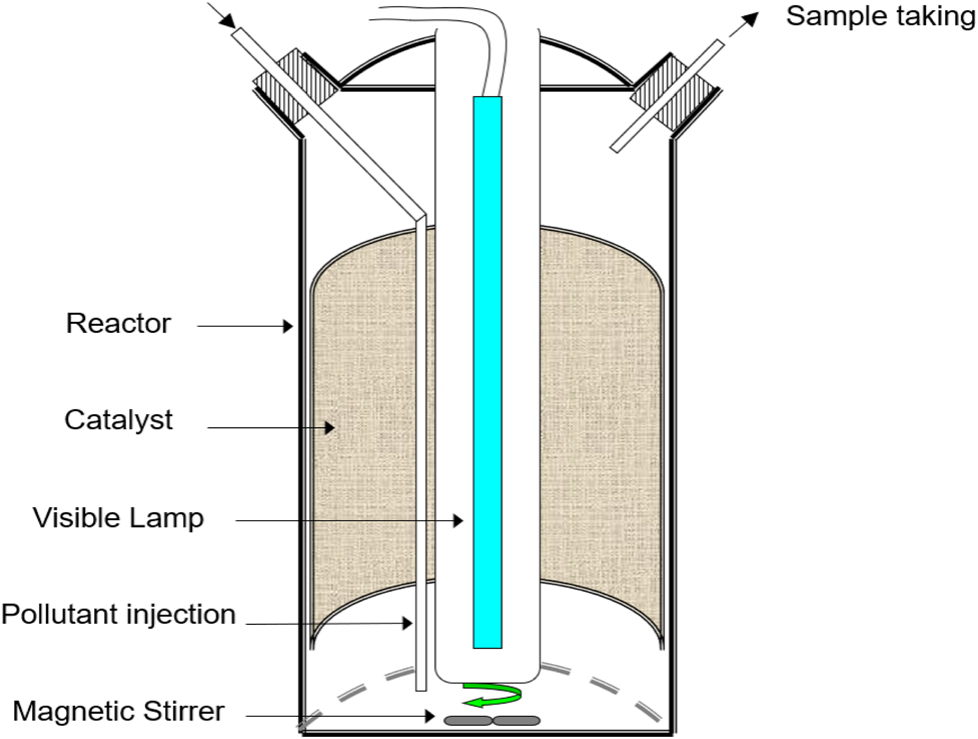
The schematic representation of the batch photoreactor featuring the Sylvania CF-L 24 W/840 coaxial central source (400–720 nm).

A specific reactor evaluated the photocatalytic activity of the PbS-NPs/TiO_2_-NTs catalyst in the degradation of Butane-2, 3-Dione (BUT: C_4_H_6_O_2_). The photocatalytic experiments were performed in a cylindrical batch reactor, as illustrated in Fig. 1. A Sylvania CF-L 24 W/840 emitting visible and near-visible light (380–720 nm),^7^ illuminated the reactor from above, positioned outside it.

We monitored the alteration in BUT concentration and the formation of oxidation by-products were monitored using a gas chromatograph (Clarus GC-500) equipped with a flame ionization detector (FID). The chromatograph featured an apolar capillary DB-MS column with a length of 60 meters and a diameter of 0.25 mm (with a film thickness of 0.25 μm). The detector (FID) operated on a mixture of air and hydrogen (H_2_), with helium (He) serving as the carrier gas at a flow rate of 1 mL min^−1^. The analysis conditions, including the oven temperature, were initially set at 50 °C for 3 minutes and subsequently programmed to increase to 100 °C at a rate of 2°C min^−1^, remaining at the maximum temperature for 10 minutes.

Injection and detection temperatures were maintained at 250 °C for both and were utilized to quantify the inlet and outlet BUT concentrations. Each catalyzed sample (BUT), comprising a volume of 500 μL, was manually injected using a gas-tight syringe, and this procedure was repeated at least three times.

## 3 Results & discussions

### 3.1. PbS NPs-decorated TiO_2_ NTs characterizations

**Fig. 2:**
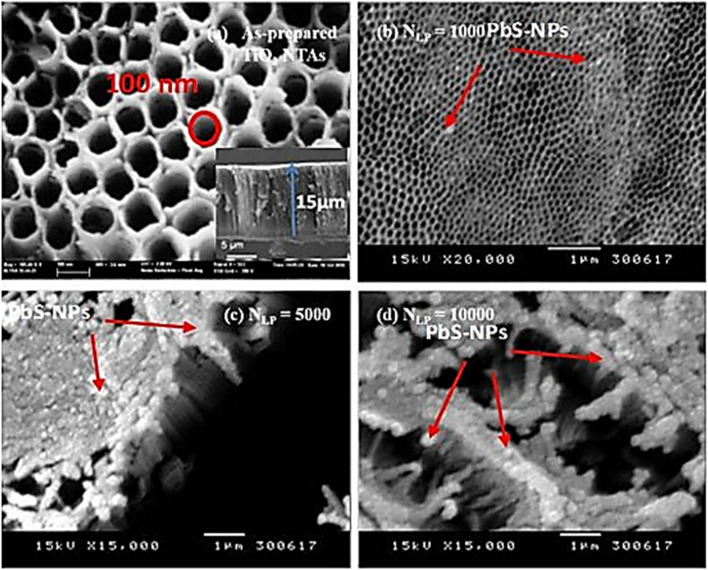
SEM top-view images depicting (a) pure TiO_2_ NTs, (b) PbS-NPs-decorated TiO_2_ NTs at *N*_LP_ = 1000, (c) *N*_LP_ = 5000, and (d) *N*_LP_ = 10 000.

FESEM top-view images in Fig. 2(a)–(d) display the surfaces of pure TiO_2_ and PbS NPs-decorated TiO_2_ NTs at different *N*_LP_ values. Fig. 2(a) shows a uniform surface with vertically aligned TiO_2_ NTs on the Ti substrate. These boast an average diameter of approximately 100 nm and a length of 15 μm. Figures 2(b) to 2(d) display SEM top-view images of the TiO_2_ surface decorated with PbS NPs, each corresponding to different laser pulses, namely *N*_LP_ = 1000, *N*_LP_ = 5000, and *N*_LP_ = 10 000.

**Fig. 3:**
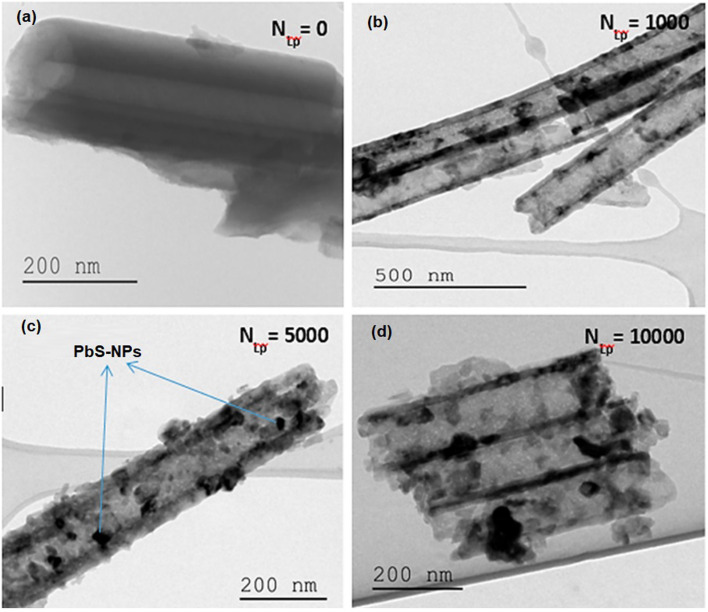
TEM images portraying PbS-NPs-decorated TiO_2_-NTs for (a) *N*_LP_ = 0, (b) *N*_LP_ = 1000, (c) *N*_LP_ = 5000, and (d) *N*_LP_ = 10 000.

The deposition of PbS nanoparticles onto the surface of TiO_2_ nanotubes has been accomplished. It is worth noting that the size and distribution of the PbS nanoparticles across the surface of TiO_2_ nanotubes depend on the number of laser pulses (*N*_LP_). Fig. 2(b) illustrates an SEM image that reveals the presence of small PbS nanoparticles scattered on the surface of TiO_2_ nanotubes. However, as the *N*_LP_ increases beyond 1000 pulses, PbS nanoparticles start to aggregate and eventually block the pores of the TiO_2_ nanotubes, as shown in Fig. 2(c) and (d). TEM analysis was performed to gain a more comprehensive understanding of the shape and distribution of the PbS nanoparticles on the surface of TiO_2_ nanotubes. The PbS nanoparticles and TiO_2_ nanotubes were carefully detached from the Ti substrates and dispersed in ethanol. The resulting mixture was then drop-cast onto copper TEM grids. The resulting TEM images of the PbS nanoparticles and TiO_2_ nanotubes, acquired with *N*_LP_ values of 1000, 5000, and 10 000, are presented in Fig. 3(b), (c), and (d), respectively.

**Fig. 4:**
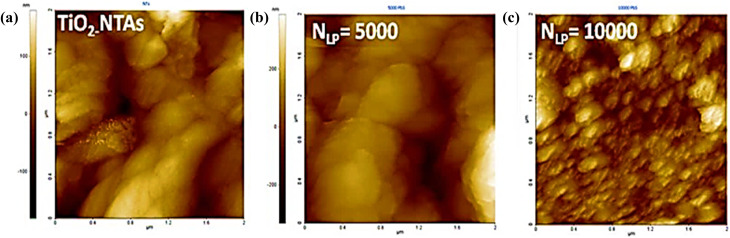
AFM (2D) scans depicting (a) TiO_2_-NTs, (b) PbS (*N*_LP_ = 5000)/TiO_2_ -NTs, and (c) PbS (*N*_LP_ = 10 000)/TiO_2_ -NTs.

The findings affirm a clear correlation between the dispersion of PbS NPs and the number of laser pulses (*N*_LP_). At lower *N*_LP_ values (*N*_LP_ = 1000 and 5000), tiny spherical PbS NPs exhibit even distribution along the walls of the nanotubes, resulting in an increased loading of PbS as *N*_LP_ rises. However, when the *N*_LP_ reaches 10 000, as depicted in Fig. 3(d), a notable particle aggregation occurs, obstructing the TiO_2_ NTs' pore entrances. For a more comprehensive assessment of the morphology of both undecorated and PbS-NPs-decorated TiO_2_-NTs samples, an atomic force microscope (AFM) analysis was conducted. The AFM surface topography of the deposited PbS-NPs/TiO_2_-NTs, as shown in Fig. 4, exhibits variations linked to the *N*_LP_. The surface morphology of the PbS demonstrates compactness, uniformity, and firm adherence to the substrate.

**Fig. 5:**
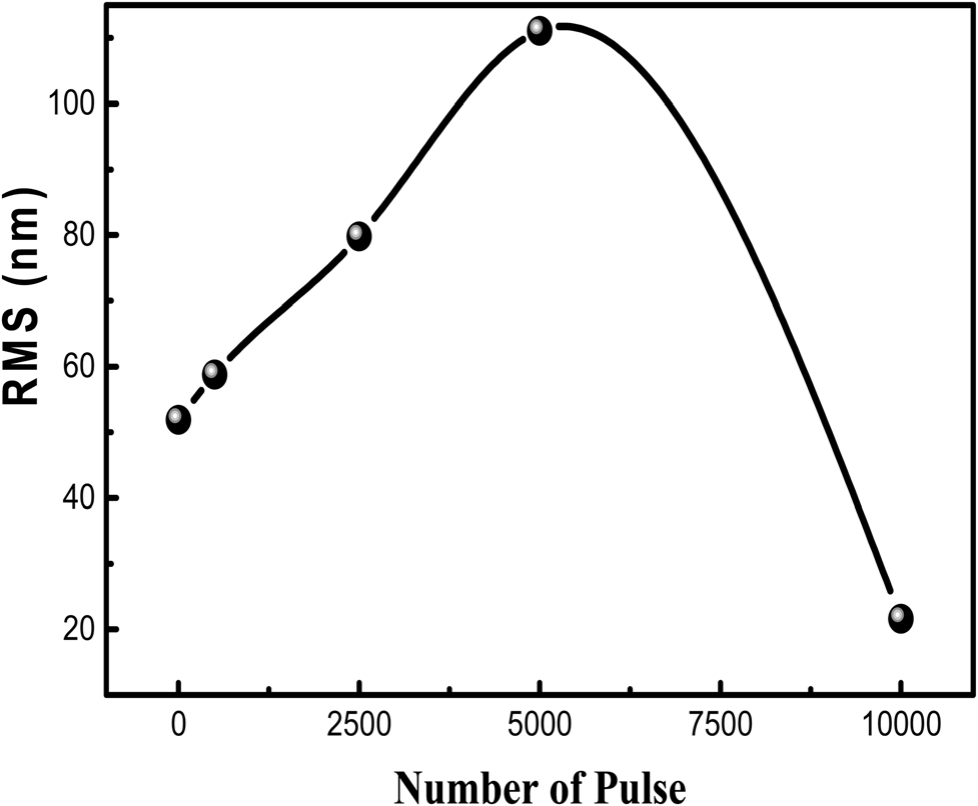
Illustrates the variation of root mean square (RMS) roughness in PbS NPs/TiO_2_ NTs samples as a function of *N*_LP_.

The surface roughness exhibits variation, with a value of 52 nm for undecorated TiO_2_ NTs (as depicted in Fig. 5). In comparison, it increases to 111 nm for *N*_LP_ = 5000 and then decreases again to 22 nm for the number of laser pulses *N*_LP_ = 10 000. This behavior can be attributed to the nucleation coalescence growth process of PbS species on the TiO_2_ surface.

**Fig. 6:**
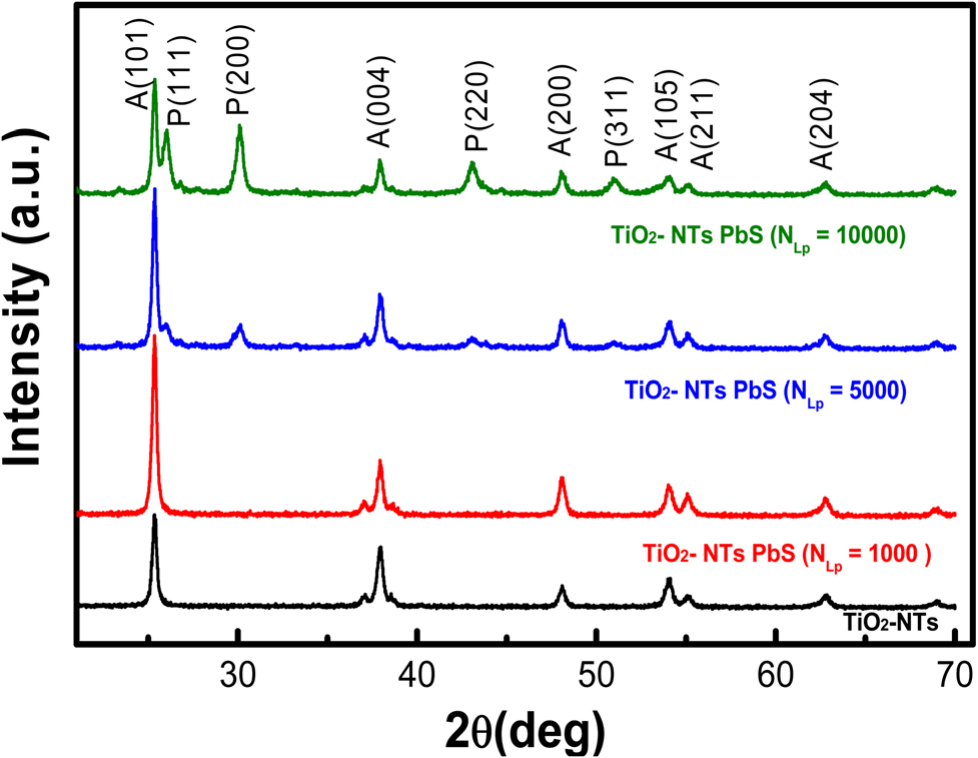
XRD patterns of pure TiO_2_ nanotubes and PbS-decorated TiO_2_ nanotubes prepared at different laser pulse numbers (*N*_LP_), with peaks from TiO_2_ and PbS labeled A and P, respectively.

Fig. 6 shows the X-ray diffraction (XRD) pattern for undecorated TiO_2_ NTs and PbS-decorated TiO_2_ NTs prepared with varying *N*_LP_ values. The diffraction peaks at 2*θ* = 25.34, 38.00, 47.99, 54.08, 55.05, and 62.83 correspond to the reflections of (101), (004), (200), (105), (211), and (204) planes of the tetragonal phase of the anatase form of TiO_2_, with no apparent impurities. The other diffraction peaks align with the face-centred cubic phase of PbS, as per JCPDS card no. 5-592. The intensification of peak maxima and the narrowing of full width at half-maximum (FWHM) are observed with an increase in *N*_LP_ from 1000 to 10 000. The crystallite average size of synthesized PbS NPs is determined using the Debye–Scherrer formula (Eq. (1)), with values ranging from 15.8 nm to 16.6 nm as *N*_LP_ increases from 5000 to 10 000. The crystallite size of PbS NPs has been determined in our previously published work (Hajjaji *et al.*, 2019).^24^

1 *D* = *kλ*/*β* cos *θ*

where *λ* is the wavelength of the incident beam (1.5405 Å), *K* is Scherrer's constant (0.9), *θ* the diffraction angle related to a given XRD peak, and *β* corresponds to the full width at half maximum of the diffraction peak.

**Fig. 7:**
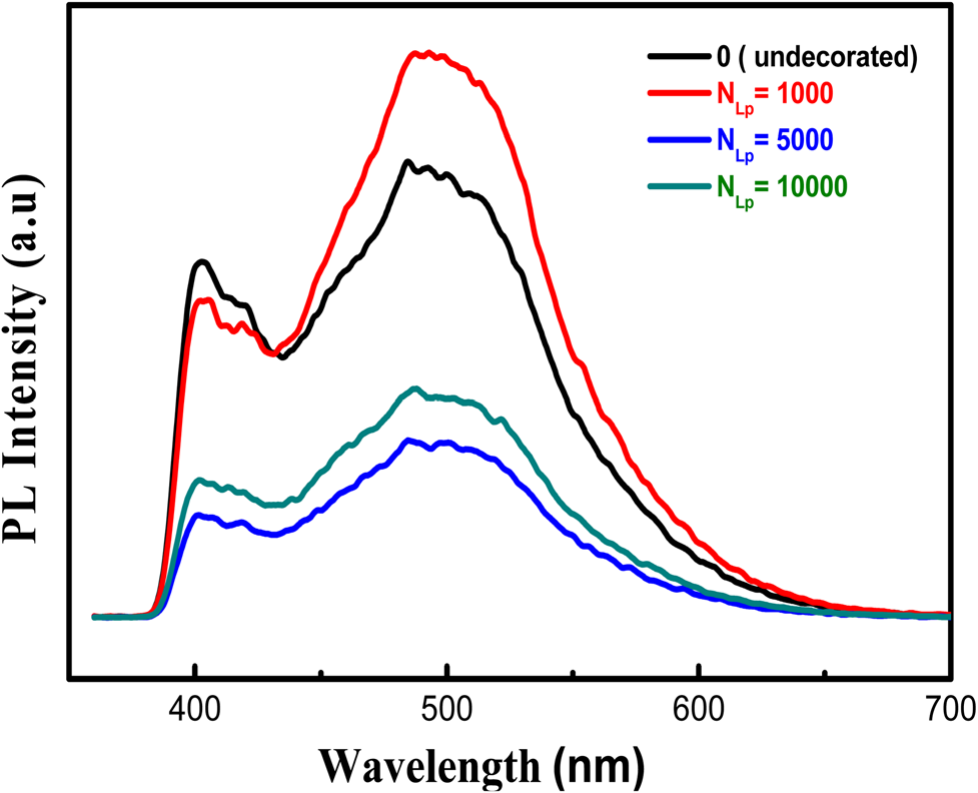
Shows the photoluminescence spectra of pure and PbS-decorated TiO_2_ nanotubes prepared with varying *N*_LP_.

Photoluminescence emission is employed to assess electron–hole recombination characteristics. Fig. 7 illustrates the photoluminescence spectra of both decorated and undecorated TiO_2_ NTs with similar peak positions, while photoluminescence intensity varies depending on *N*_LP_. Lower photoluminescence intensity implies a reduced electron–hole recombination rate and an extended lifetime of photogenerated carriers.

It's evident that, for *N*_LP_ values of 5000 and 10 000, PbS-NPs/TiO_2_-NTs exhibit lower photoluminescence intensity than undecorated NTs, with the lowest intensity observed at *N*_LP_ = 5000. This phenomenon can be attributed to a surface and an efficient electron transfer between the PbS NPs and the TiO_2_ NTs, reducing electron–hole recombination rates. Furthermore, previous studies, such as that by Pandit *et al.*^35^ on TiO_2_-based hybrid nanosystems, have shown that emission peaks around 550 nm are often associated with oxygen vacancy-related defects, which act as trapping centers for excitons. Although our spectator does not show a pronounced peak at 550 nm, the variation in intensity as a function of *N*_LP_ reflects the influence of the size and distribution of PbS NPs on recombination processes. As *N*_LP_ increases beyond 5000, PbS NPs aggregate, increasing particle sizes. The increase in PL intensity for *N*_LP_ = 10 000, due to NPs aggregation, suggests that additional defects or increased radiative recombination may occur when the surface morphology is changed.

**Fig. 8:**
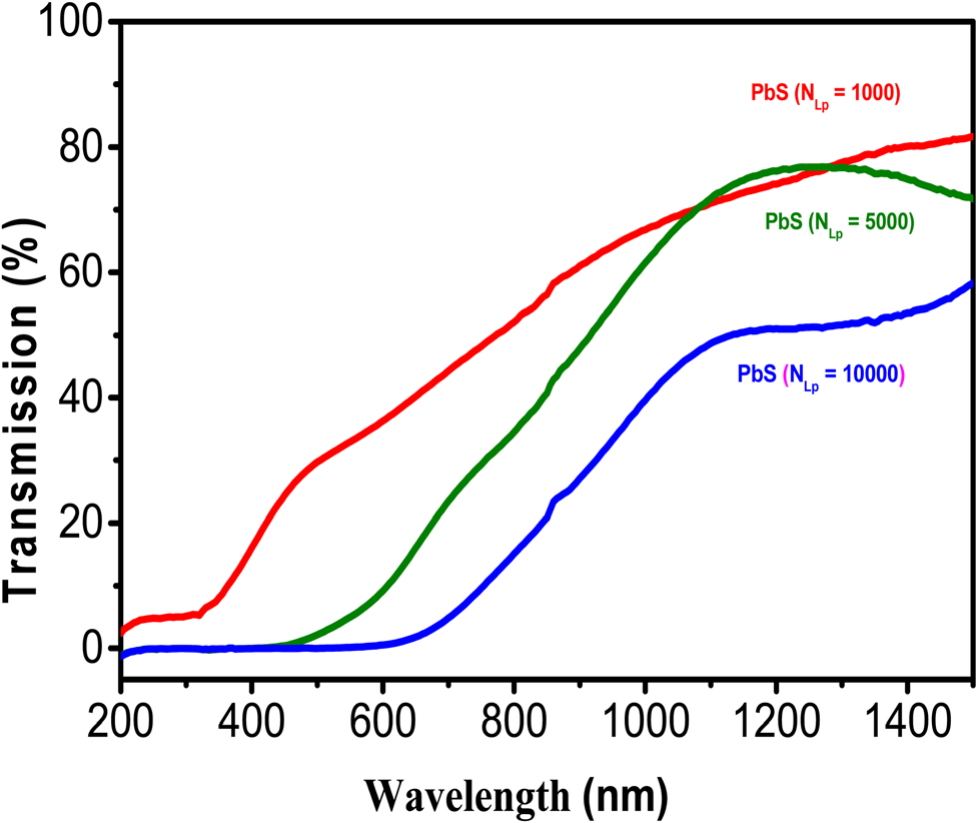
Displays the transmission spectra of PbS-NPs deposited on quartz substrates for different *N*_LP_ values.

The transmittance spectrum of PbS NPs on a quartz substrate is presented in Fig. 8. An observable redshift (towards longer wavelengths) is noted as *N*_LP_ increases, indicating that the loading of PbS NPs, as *N*_LP_ rises, increases NP size, thereby enhancing visible light absorption. This shift results from the decrease in the PbS bandgap as the size of PbS-NPs increases, approaching the bulk value (0.41 eV) with increasing *N*_LP_.

The optical energy band gap of PbS NPs is estimated using Tauc's relation, which relies on the measured transmittance spectra data and involves several vital parameters.

2 (*αhv*)^2^ = *A*(*hv* − *E*_g_)^*n*^/*hv*

In this relation, *α* represents the absorption coefficient, *h* stands for Planck's constant, *v* denotes the frequency of the incident light, *A* is a constant interrelated with the effective masses of electrons and holes, as well as the refractive index, *hv* signifies the photon energy, *E*_g_ represents the energy band gap of the material, and *n* is a constant that depends on the nature of the optical transition. Specifically, *n* takes values of 1/2 and 2 for direct and indirect band gap transitions, respectively. The band gap energy (*E*_g_) is determined by drawing a line from the maximum slope of the curve to the *x*-axis, as depicted in Fig. 8.

The band gap energy value, which estimates the optical energy band gap of PbS NPs, is determined from the measured transmittance spectra data using Tauc's relation. Band gap values for PbS NPs prepared under different *N*_LP_ conditions (1000, 5000, and 10 000 pulses) were estimated from the Tauc plot. These values exhibit a decreasing trend with increasing *N*_LP_, as shown in Table 2.

**Table 2:** Optical band gap values for PbS NPs were prepared using different *N*_LP_ values

*N*_LP_ (pulses)	1000	5000	10 000
Optical and gap (eV)	1.25	1.08	0.93

It is essential to highlight that our work has previously reported the optical band gap value for TiO_2_ NTs (3.1 eV).^24^

**Fig. 9:**
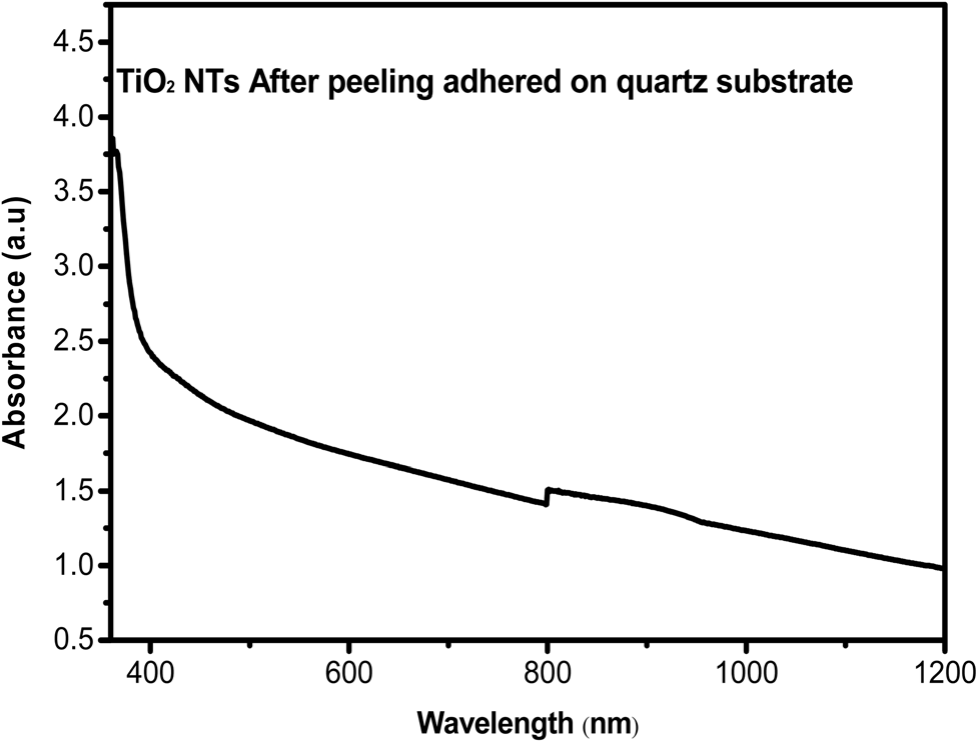
Absorbance spectra of TiO_2_ NTAs after peeling and adhering to quartz substrate.

Additionally, Fig. 9 displays the absorbance spectra of TiO_2_ NTAs after peeling and adhering to a quartz substrate. These spectra offer valuable insights into the optical properties of the TiO_2_ nanotube arrays.

### 3.2. Photocatalytic experiments

**Fig. 10:**
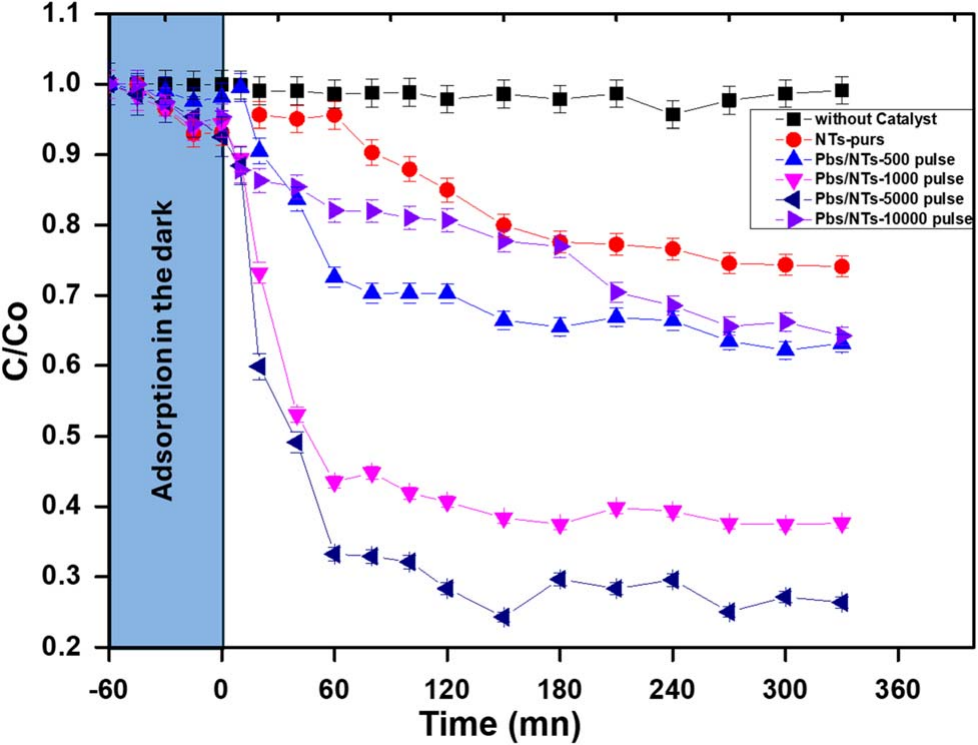
The plot of absorbance *versus* irradiation time, using PbS-NPs/TiO_2_-NTs samples, prepared different pulses.

The efficient photocatalytic performance of obtained catalysts can be primarily attributed to the energy levels of the two semiconductors, as illustrated in Fig. 10. This energy level alignment results in an improved photo response in the visible light spectrum, effectively reducing the recombination of electron–hole pairs.

The figure illustrates the photocatalytic degradation performance of various PbS-NPs/TiO_2_-NTs catalysts. Before activating the lamp, the assembly is left in the dark for one hour to establish an adsorption–desorption equilibrium between the butane-2,3-dione (BUT) pollutant and the catalyst. The initial adsorption phase in the dark (blue zone) is followed by subsequent photocatalytic activity under irradiation. While the control sample (without a catalyst) maintains a nearly constant *C*/*C*_0_ ratio of around 1.0, the modified samples exhibit varying levels of photocatalytic performance. The degradation efficiency systematically increases with the pulse number, with pure NTs demonstrating modest activity and samples subjected to higher pulse numbers (5000 and 10 000) achieving the most significant degradation, reaching approximately 70%. This enhanced performance can be attributed to the optimal alignment of energy levels between the two semiconductors (PbS and TiO_2_), effectively improving the response to visible light and reducing electron–hole pair recombination. The pulse number is critical in optimizing the semiconductor junction interface for maximum photocatalytic effect.

**Fig. 11:**
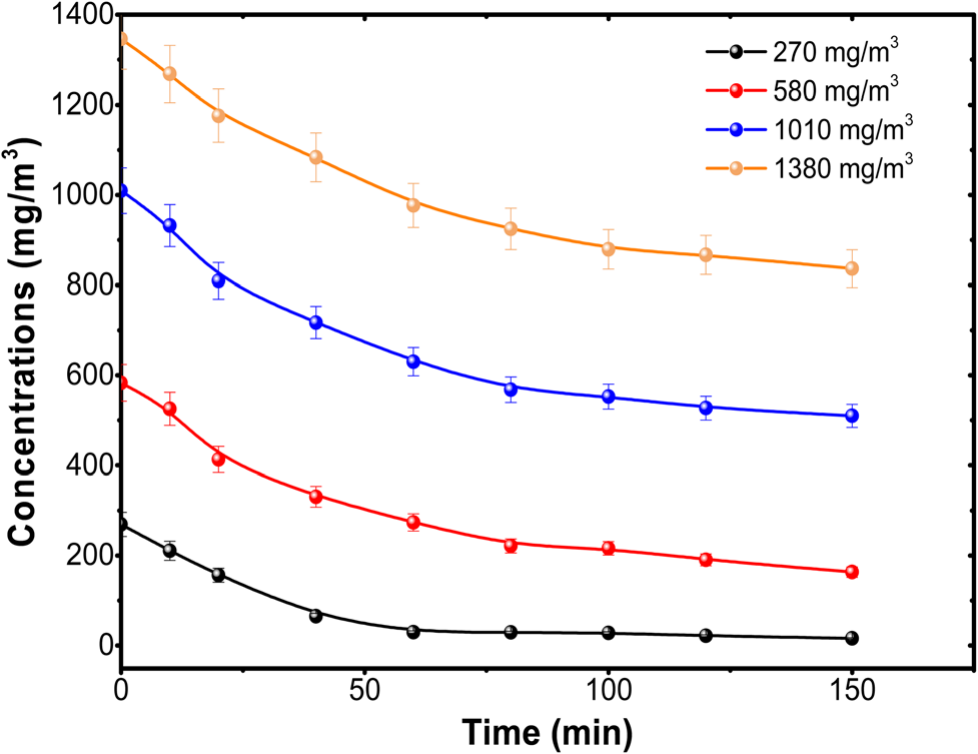
Variation of the concentration of BUT using 5000 pulse PbS-NPs/TiO_2_-NTs at different inlet concentrations.

Fig. 11 reveals the BUT concentration over time when using the 5000 pulse PbS-NPs/TiO_2_-NTs catalyst at varying initial BUT concentrations. A notable observation is that the initial degradation rate (*t* = 0) decreases as the initial BUT concentration at the inlet increases. This trend is due to the higher availability of photocatalytic sites when the initial concentration is lower,^36^ as a higher initial concentration does not proportionally increase the number of molecules participating in the reaction, leading to a decrease in degradation efficiency.

**Fig. 12:**
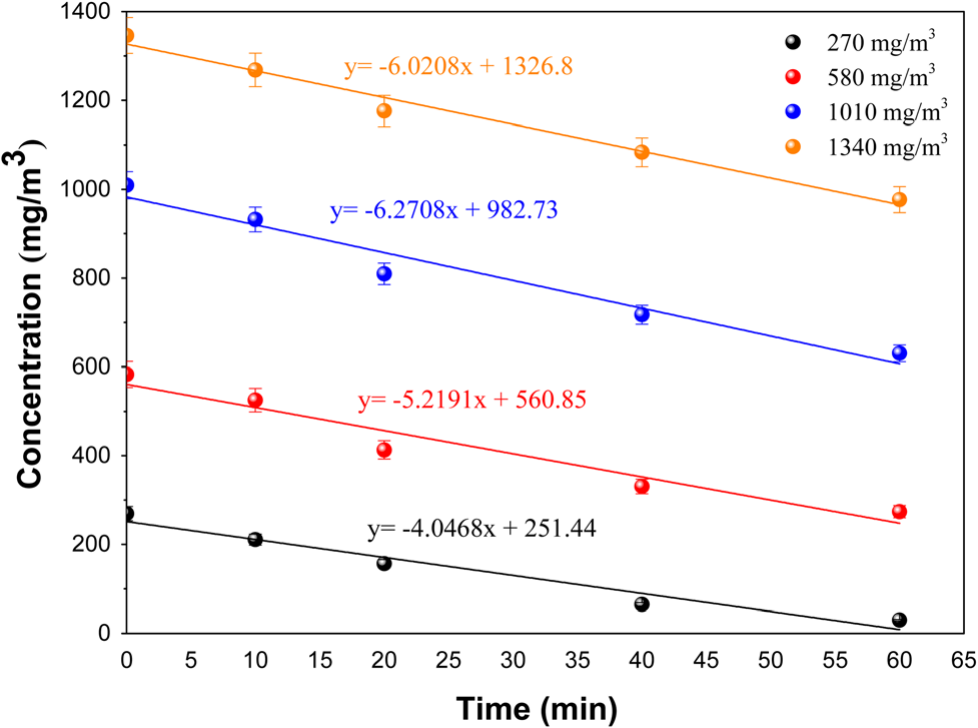
The plot of the first part (60 min) of butadiene degradation determines the initial degradation rate.

In describing the photocatalytic performance of the 5000 pulse PbS-NPs/TiO_2_-NTs, we employed the Langmuir–Hinshelwood (L–H) model equation:^36^Fig. 12 shows the plot of the first part (60 min) of butadiene degradation determines the initial degradation rate.3
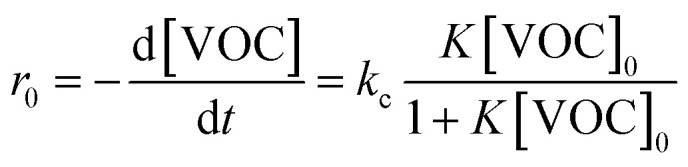
Here, *r*_0_ (mg (m^−3^ min^−1^)) represents the initial photocatalytic degradation rate, [VOC] is the initial BUT concentration (mg m^−3^), *K* is the adsorption constant, and kc is the kinetic constant under the experimental conditions.

**Fig. 13:**
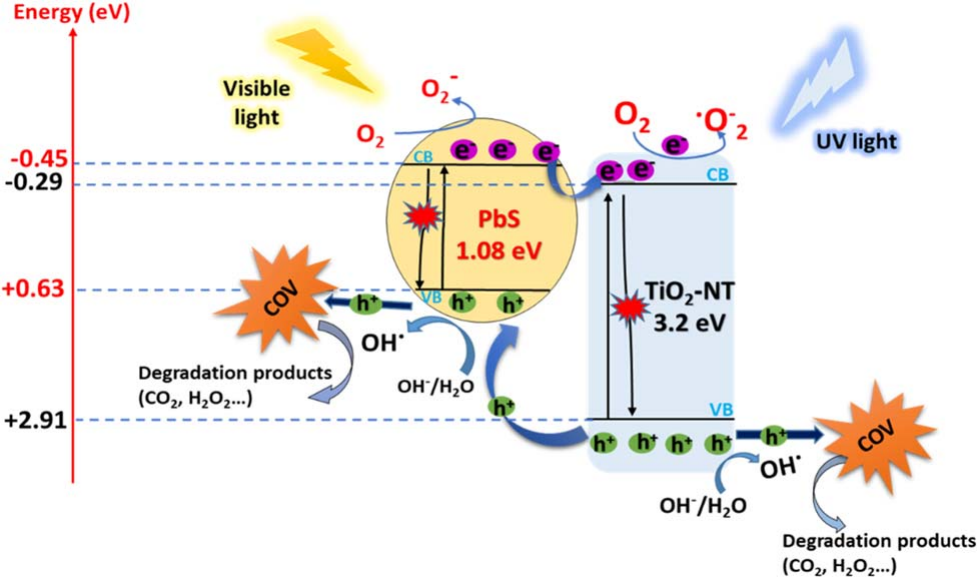
Illustration of band diagrams and the mechanism of transfer of electrons in PbS/TiO_2_-NTs nanocomposites.

Fig. 13 shows the linearized plot for the Langmuir–Hinshelwood model (L–H), which allows us to determine *k*_c_ and *K* values.4
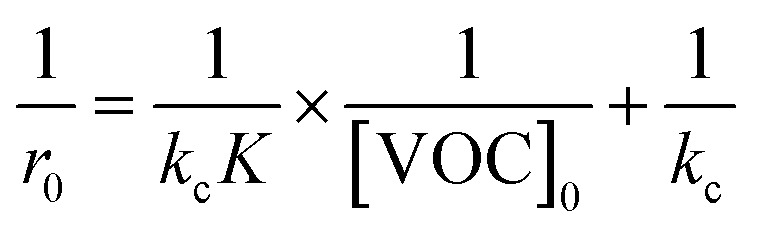


The kinetic and adsorption constants obtained from the Langmuir–Hinshelwood model are summarized in Table 3, demonstrating that the 5000 pulse PbS-NPs/TiO_2_-NTs catalyst exhibits a faster BUT removal rate in comparison to other catalysts like TiO_2_ impregnated polyester (PES) and glass fiber (GFT)-TiO_2_ (GFT-TiO_2_, PES-TiO_2_).^37–41^Table 3 displays the kinetic and adsorption constants derived from the Langmuir–Hinshelwood model (L–H) for the 5000 pulse PbS-NPs/TiO_2_-NTs catalyst.

**Table 3:** L–H constants (*k*_c_ and *K*) on 5000 pulse PbS-NPs/PS/c-Si NTs catalyst

*k*_c_: kinetic constant of L–H (mg m^−3^ min^−1^)	*K*: adsorption constant of L–H (m^3^ g^−1^)
7.24	4.7

Following a comprehensive analysis of the provided information, we can now delve deeper into understanding the degradation mechanism of BUT on PbS/TiO_2_-NTs photocatalysts, as illustrated in Fig. 13. To fully comprehend this process, it is essential to consider the energy levels of the valence band (VB) and conduction band (CB). It is interesting to note that TiO_2_ boasts a VB level with a potential of approximately +2.91 V (relative to the Normal Hydrogen Electrode, NHE), which is notably more favorable than PbS with a VB potential of +0.63 eV (relative to NHE). Equally significant is the maximum CB potential of TiO_2_, which stands at −0.29 V (relative to NHE), making it considerably more reductive than PbS, with a CB potential of −0.45 eV (relative to NHE) for *N*_LP_ = 5000.^42^ This band configuration creates a thermodynamic driving force of 0.16 eV for electron transfer from the PbS CB to the TiO_2_ CB, explaining the improved charge separation observed in our photoluminescence results.

When these photocatalysts are exposed to light, fascinating events unfold. The illumination causes the excitation of TiO_2_ nanotubes, leading to the generation of hole electrons within these nanotubes.^35,43^ This process becomes even more interesting when we consider the unique VB and CB positions of PbS and TiO_2_. Specifically, the photoexcited electrons (e^−^) in the CB of PbS-NPs exhibit a remarkable ability to rapidly migrate to the TiO_2_-NTs under UV-visible illumination. This electron transfer mechanism is consistent with the behavior observed by Pandit *et al.* in their study on Methylene Blue degradation over a PQ/TiO_2_ coupled photocatalyst, where efficient interfacial charge transfer suppressed recombination.^44^ This swift transfer is the key to enhancing the separation of photogenerated electrons and holes, effectively inhibiting their recombination tendency. In this electrons dance, the photoexcited electrons migrate from the CB of PbS-NPs to the CB of TiO_2_-NTs.

Here, an exciting interaction occurs as these transferred electrons react with absorbed O_2_, ultimately contributing to the formation of ˙O^−^_2_ radicals, which confirmed by Pandit *et al.* in their research on the photocatalytic activity of micro/nano-α-Fe_2_O_3_.^45^ These radicals have a key role in the process, generating various oxidizing radicals, including ˙OH. These ˙OH radicals result from the interaction of superoxide radicals and H^+^ ions.

In summary, this complex interaction between energy levels and electron migrations within the PbS/TiO_2_-NTs photocatalysts drives the degradation of BUT. This elegant process enables efficient photocatalysis and promises a brighter future for harnessing solar energy for environmental applications. The photocatalytic process is summarized by eqn (5a–h):

5.a PbS/TiO_2_ + *hv* → PbS/TiO_2_ (e^−^) + PbS/TiO_2_ (h^+^)

5.b e^−^ + O_2_ → ˙O^−^_2_

5.c h^+^ + H_2_O → ˙OH + H^+^

5.d 4 h^+^ + 2H_2_O → 4H^+^ + O_2_

5.e OH + BUT → degradation products

5.f ˙O_2_^−^ + BUT → degradation products

5.g h^+^ + BUT → degradation products

5.h e^−^ + BUT → degradation products

These reactions contribute to the efficient photocatalytic degradation of BUT on the PbS/TiO_2_-NTs nanocomposites, and the band diagrams in Fig. 13 illustrate the mechanism of electron transfer in these nanocomposites.

While degradation by-products were not directly measured in this study, our previous work on butadione degradation using TiO_2_-based photocatalysts decorated with silver NPs^46^ identified specific by-products, including acetone (C_3_H_6_O), acetaldehyde (C_2_H_4_O), and acetic acid (C_2_H_4_O_2_). These findings demonstrate that BUT mineralization occurs through successive oxidation steps, during which the dicarbonyl structure undergoes C–C bond cleavage and sequential hydroxylation before final conversion to CO_2_ and H_2_O. This mechanism suggests a complete mineralization of the pollutant, which is crucial for environmental remediation applications.

Although butane-2,3-dione was used as a model VOC in this study, our previous work on TiO_2_-based nanocomposites confirms that the same ˙OH radical-based pathways extend to other species.^45–47^ Aldehydes (formaldehyde, acetaldehyde) degrade more easily due to their lower oxidation potential, while aromatic compounds (*e.g.*, toluene) require longer irradiation but benefit from the improved visible light absorption of PbS-modified TiO_2_. Regarding durability, although no reusability tests were performed here, the strong adhesion of PbS to TiO_2_, confirmed by AFM, combined with the known photochemical stability of both oxides, suggests promising stability.

## 4 Conclusions

PbS-NPs-decorated TiO_2_ nanotubes were investigated in our study and synthesized with varying nanosecond laser pulses (*N*_LP_). This research has revealed that these nanocomposites hold great potential for solar-driven environmental applications. Enhanced photocatalytic performance is achieved due to the unique band alignment between PbS and TiO_2_. Efficient separation of photogenerated electrons and holes by PbS NPs, reducing their recombination, is observed. Notably, this effect results in a significant enhancement of photocatalytic efficiency, particularly in visible light, with an efficiency greater than 75% for BUT degradation at an optimal *N*_LP_ = 5000. The average diameter of PbS-NPs increased from approximately 10 nm to 20 nm with increasing *N*_LP_, and the roughness increased from 52 nm for pure TiO_2_-NTs to 111 nm for *N*_LP_ = 5000, then decreased to 22 nm for *N*_LP_ = 10 000. The analysis of optical properties demonstrates a decrease in the band gap of the nanocomposites as *N*_LP_ increases, leading to improved visible light absorption, with the lowest PL observed at the optimal *N*_LP_, indicating enhanced electron–hole recombination.

Furthermore, the potential for practical applications is emphasized by the Langmuir–Hinshelwood model, which demonstrates that the 5000 pulse PbS-NPs/TiO_2_-NTs catalyst outperforms alternative options in butadiene degradation. The efficient degradation of butadiene is facilitated by the photocatalytic mechanism, which highlights the role of energy band positions in the TiO_2_–PbS nanocomposites. These findings suggest that these nanocomposites offer promise for solar-driven environmental solutions. These materials' unique properties and efficient performance contribute to the ongoing research in photocatalysis. Hopefully, this work will inspire further exploration and application of these materials in real-world environmental remediation scenarios.

## Ethics approval and consent to participate

This study does not involve any experiments with human participants or animals).

## Author contributions

Mabrouk Abidi and Amine Aymen Assadi: Conceptualization, data curation, Mabrouk Abidi: Writing – original draft, Safa Jemai, Anouar Hajjaji, Mounir Gaidi, Achraf Amir Assadi, Nabisab Mujawar Mubarak and My Ali El Khakani: Writing – review & editing. All authors have read and agreed to the published version of the manuscript.

## Conflicts of interest

The authors declare that they have no competing interests.

## Funding statement

This work was supported and funded by the Deanship of Scientific Research at Imam Mohammad Ibn Saud Islamic University (IMSIU) (grant number IMSIU-DDRSP2502).

## Data Availability

The data presented in this study are available in the manuscript.

## References

[cit1] Anitha V. C., Goswami A., Sopha H., Nandan D., Gawande M. B., Cepe K., Macak J. M. (2018). Appl. Mater. Today.

[cit2] Pradhan S. K., Reucroft P. J., Yang F., Dozier A. (2003). J. Cryst. Growth.

[cit3] Pérez-Jiménez L. E., Solis-Cortazar J. C., Rojas-Blanco L., Perez-Hernandez G., Martinez O. S., Palomera R. C., Morales E. R. (2019). Results Phys..

[cit4] Sun Y., Liu E. D., Zhu L., Wen Y., Tan Q. W., Feng W. (2019). Dig. J. Nanomater. Biostructures.

[cit5] Naduvath J. , Bhargava P. and Mallick S., in AIP Conference Proceedings, AIP Publishing LLC, 2019, vol. 2082, p. 030019

[cit6] Liang Y., Guan Z. C., Wang H. P., Du R. G. (2017). Electrochem. Commun..

[cit7] Macak J. M., Tsuchiya H., Schmuki P. (2005). Angew. Chem., Int. Ed..

[cit8] Tang C., Huang X., Wang H., Shi H., Zhao G. (2020). J. Hazard. Mater..

[cit9] Tenkyong T., Mary J. S. S., Praveen B., Pugazhendhi K., Sharmila D. J., Shyla J. M. (2018). Mater. Sci. Semicond. Process..

[cit10] Gharib-Abou Ghaida S., Assadi A. A., Costa G., Bouzaza A., Wolbert D. (2016). Chem. Eng. J..

[cit11] Yin B., Qian Q., Xiong Z., Jiang H., Lin Y., Feng D. (2019). Nanotechnology.

[cit12] Kavitha S. , Nivedita R. S., John A. and Lakshmi M., in AIP Conference Proceedings, AIP Publishing LLC, 2019, vol. 2082, p. 050007

[cit13] Arkusz K., Paradowska E., Nycz M., Krasicka-Cydzik E. (2018). J. Nanosci. Nanotechnol..

[cit14] Kim W. T. and Choi W. Y., in Applied Mechanics and Materials, 2017, vol. 864, pp. 212–217

[cit15] Schmuki P. , Liu N. and Altomare M., in Meeting Abstracts: the Electrochemical Society, 2019, vol. 43, pp. 2061–2061

[cit16] Zghab E., Hamandi M., Dappozze F., Kochkar H., Zina M. S., Guillard C., Berhault G. (2020). Mater. Sci. Semicond. Process..

[cit17] Çırak B. B., Caglar B., Kılınç T., Karadeniz S. M., Erdoğan Y., Kılıç S., Çırak Ç. (2019). Mater. Res. Bull..

[cit18] Yang X., Chen Z., Zhou D., Zhao W., Qian X., Yang Q., Shen C. (2019). Sol. Energy Mater. Sol. Cells.

[cit19] Gogoi D., Namdeo A., Golder A. K., Peela N. R. (2020). Int. J. Hydrogen Energy.

[cit20] Photharin S., Tipparach U. (2017). Int. J. Appl. Eng. Res..

[cit21] Gaidi M., Trabelsi K., Hajjaji A., Chourou M. L., Alhazaa A. N., Bessais B., El Khakani M. A. (2017). Nanotechnology.

[cit22] Trabelsi K., Hajjaji A., Gaidi M., Bessais B., El Khakani M. A. (2017). J. Appl. Phys..

[cit23] Hajjaji A., Jemai S., Rebhi A., Trabelsi K., Gaidi M., Alhazaa A. N., Bessais B. (2020). J. Mater.

[cit24] Hajjaji A., Jemai S., Trabelsi K., Kouki A., Assaker I. B., Ka I., El Khakani M. A. (2019). J. Mater. Sci.: Mater. Electron..

[cit25] Abidi M., Assadi A. A., Bouzaza A., Hajjaji A., Bessais B., Rtimi S. (2019). Appl. Catal., B.

[cit26] Khaliq N., Rasheed M. A., Cha G., Khan M., Karim S., Schmuki P., Ali G. (2020). Sens. Actuators, B.

[cit27] Khezami L., Lounissi I., Hajjaji A., Guesmi A., Assadi A. A., Bessais B. (2021). J. Mater.

[cit28] Karoui S., Assadi A. A., Amina M., Bouzaza A. (2024). Food Biosci..

[cit29] ADEME , Pollution Olfactives : Origine, Législation, Analyse, Traitement. Ademe, Dunod, Paris, 2005

[cit30] Schiavon M., Vincenzo T., Casazza A., Ragazzi M. (2017). Water, Air, Soil Pollut..

[cit31] Sultana S., Vandenbroucke A. M., Leys C., De Geyter N., Morent R. (2015). Ctalysts.

[cit32] Rodrigues C. C., de Moraes Jr. D., Nobrega S. W., Barboza M. G. (2007). Bioresour. Technol..

[cit33] Doggali P., Teraoka Y., Mungse P., Shah I., Rayalu S., Labhsetwar N. (2012). J. Mol. Catal. A:Chem..

[cit34] Hu Q., Wang C., Huang K. (2015). Chem. Eng. J..

[cit35] Pandit V., Arbuj S., Hawaldar R., Kshirsagar P., Mulik U., Gosavi S., Kale B. (2015). J. Mater. Chem. A.

[cit36] Abidi M., Hajjaji A., Bouzaza A., Trablesi K., Makhlouf H., Rtimi S., Assadi A. A., Bessais B. (2020). J. Photochem. Photobiol., A.

[cit37] Abdelkader M., Assadi A. A., Guiza M., Elfalleh W., Khezami L., Tahraoui H., Baaloudj O., Mouni L., Zhang J., Amrane A. (2025). Catalysts.

[cit38] Assadi A. A. (2024). Materials.

[cit39] Belkessa N., Assadi A. A., Tri Ph. N., Bouzaza A. (2025). Catal. Today.

[cit40] Assadi A. A., Bouzaza A., Wolbert D. (2015). J. Photochem. Photobiol., A.

[cit41] Zadi T., Nasrallah A. A. A., Bouallouche R., Tri P. N., Bouzaza A., Azizi M. M., Maachi R., Wolbert D. (2018). Chem. Eng. J..

[cit42] Zhang H., Gao Y., Zhu G., Li B., Gou J., Cheng X. (2019). Sep. Purif. Technol..

[cit43] Jawale V., Gugale G., Chaskar M., Pandit S., Pawar R., Suryawanshi S., Arbuj S. (2021). J. Mater. Res..

[cit44] Pandit V. U., Arbuj S. S., Pandit Y. B., Naik S. D., Rane S. B., Mulik U. P., Kale B. B. (2015). RSC Adv..

[cit45] Pandit V. R. U., Jadhav G. K. P., Jawale V. M. S., Dubepatil R., Gurao R., Late D. J. (2024). RSC Adv..

[cit46] Abidi M., Abou Saoud W., Bouzaza A., Hajjaji A., Bessais B., Wolbert D., Rtimi S. (2023). J. Photochem. Photobiol., A.

[cit47] Serhane Y., Belkessa N., Bouzaza A., Amrane A., Assadi A. A. (2025). Sep. Purif. Technol..

